# Kinetics of α-glucosidase inhibition by different fractions of three species of Labiatae extracts: a new diabetes treatment model

**DOI:** 10.1080/13880209.2017.1306569

**Published:** 2017-04-03

**Authors:** Sahere Rouzbehan, Soheila Moein, Ahmad Homaei, Mahmood Reza Moein

**Affiliations:** a Molecular Medicine Research Center, Hormozgan Health Institute, Hormozgan University of Medical Sciences, Bandar Abbas, IR Iran;; b Biochemistry Department, Faculty of Medicine, Hormozgan University of Medical Sciences, Bandar Abbas, IR Iran;; c Department of Biochemistry, Faculty of Science, University of Hormozgan, Bandar Abbas, IR Iran;; d Medicinal Plants Processing Research Center, Shiraz University of Medical Sciences, Shiraz, IR Iran;; e Department of Pharmacognosy, School of Pharmacy, Shiraz University of Medical Sciences, Shiraz, IR Iran

**Keywords:** Enzyme retardation, *Zataria multifolra*, *Salvia mirzayanii*, *Otostegia persica*

## Abstract

**Context:** Glucosidases are a group of enzymes playing crucial roles in digestion of carbohydrates. Glucosidase inhibitors can reduce carbohydrate digestion rate and have the potential to prevent development of type 2 diabetes. The Labiatae is one of the largest plant families grown globally and many studies that have isolated new pharmaceutical compounds. In folk medicine, some of Labiatae plants such as *Zataria multiflora* Boiss, *Salvia mirzayanii* Rech. F. & Esfand, and *Otostegia persica* Boiss are consumed for the treatment of diabetes.

**Objectives:** This study investigates the inhibitory effects of different fractions of three mentioned species extracts on α-glucosidase.

**Materials and methods:** Ethanol extracts of these plants leaves were fractionated using petroleum ether, chloroform, ethyl acetate, and *n*-butanol solutions. The duration of this study was 12 months. To measure enzyme inhibition, 5 μL of the enzyme, 20 μL of substrate and samples were used and for evaluation mode of inhibition, constant amounts of α-glucosidase were incubated with rising concentrations of substrate (PNPG).

**Results:** The results revealed that the ethyl acetate fraction of *Zataria multiflora* (IC_50_ = 0.35 ± 0.01 mg/mL) and petroleum ether fraction of *Salvia mirzayanii* (IC_50_ = 0.4 ± 0.11 mg/mL) were the most potent inhibitors of α-glucosidase in comparison with the other samples and acarbose as the standard (IC_50_ = 7 ± 0.19 mg/mL). All of the samples exhibited noncompetitive-uncompetitive inhibition.

**Discussion and conclusion:** It can be inferred from this study that α-glucosidase inhibitory potential of the studied extracts may be a marker of antidiabetic potential of these extracts.

## Introduction

Prevalence of type 2 diabetes mellitus (T2DM) has been increasing rapidly over the past decade. Unless appropriate action is taken, it is foretold that there will be at least 350 million people in the world with type 2 diabetes by the year 2030 (World Health Organization 2003). Type 2 diabetes mellitus (T2DM) is a multifaceted heterogeneous group of metabolic disarray with hyperglycemia and defective insulin action and/or insulin secretion (Lin & Sun 2010). Impairment of insulin actions is known as insulin resistance, the failure which is located at the signaling pathways held after insulin binding to its receptor.Chronic occurrence of the mentioned failure leads to hyperglycemia (Fernández-Mejía 2013). Postprandial hyperglycemia is a prominent and early defect in diabetes type 2, which can induce oxidative stress through excessive generation of free radicals that may impair the endogenous antioxidant defense and in turn lead to various secondary complications including cardiovascular risk factors (Johansen et al. 2005). Therefore, one of the therapeutic approaches proposed for diabetes is to control the postprandial hyperglycemia by delaying glucose absorption (Akhilesh 2012). One of the strategies adopted to treat diabetes mellitus is inhibition of carbohydrate breakdown enzymes such as α-glucosidase (EC 3.2.1.20) in the epithelial mucosa of small intestine performed with association retardation of intestinal glucose absorption and decrease of postprandial blood glucose levels (Shai et al. 2010). Nowadays, some α-glucosidase inhibitors, e.g., acarbose and voglibose, are widely used clinically to control patients blood glucose levels. Although they are observed to be effective, they usually cause negative effects such as abdominal distension, flatulence, meteorism, and possibly diarrhea (Verspohl 2012). With consideration of the mentioned point, many efforts have been made to specify more effective and unique α-glucosidase inhibitors, and a good number of them have been identified with application of nature sources. On account of their matchless chemical diversity and biological connection, natural materials have been widely recognized as potential chemical resources in drug screening. Therefore, discovery of α-glucosidase inhibitors from natural materials for treatment of diabetes can be regarded as a promising topic (Coman et al. 2012). To treat T2DM, more than 1000 plant species have been made use of worldwide (Trojan-Rodrigues et al. 2012). Lamiaceae family is one of the largest families of flowering plants with approximately 220 genera and almost 4000 species. Several species of this family such as *Zataria multiflora* Boiss (*Zm)*, *Salvia mirzayanii* Rech. F& Esfand (*Sm*), and *Otostegia persica* Boiss (*Op*) (Naghibi et al. 2010) are used to treat diabetes in both traditional and modern medicine. Studies conducted in this regard have demonstrated that *Zm* aqeous extract has a strong inhibitory effect on α-glucosidase (Gholamhoseinian et al. 2008) and also significantly decreases postprandial glucose level (Najar et al. 2009). This extract increases insulin sensitivity and PPARγ gene expression in high fructose fed insulin resistant rats (Mohammadi et al. 2014). The aerial parts of *Sm* are used in Iranian traditional medicine as anti-stomachache, anti-diabetes, and anti-spasmolytic (Keller 1978; Zargari 1990; Kamatou et al. 2008). *Otostegia persica* is traditionally used in some regions of Iran as antidiabetic medicinal plant. Thus, its addressed potential has been investigated by some research groups (Lima et al. 2006; Ebrahimpoor et al. 2009; Tofighi et al. 2009; Hajzadeh et al. 2011; Manzari-Tavakoli et al. 2013; Mohammadi et al. 2014; Sepehri et al. 2014). As the review literature reveals no documents about the effects of different fractions of *Zm*, *Op*, and *Sm* extracts on inhibition of α-glucosidase have been presented. Hence, the very aim of this study was to investigate this property.

## Materials and methods

### Chemicals

PNPG (4-nitrophenyl α-d-glucopyranoside) and α-glucosidase from *Saccharomyces cerevisiae* were purchased from Sigma Aldrich, USA. Other chemicals were purchased from Merck. 

### Specimen collection and extracts fractionation

In this study, 1 kg of aerial parts of three plants was used. *Sm (*MPRCM 94-84) and *OP* (MPRCM 94-86) were collected from Genu Mountains in the northeast of Bandar Abbas on 20 and 15 June, 2012, respectively. *Zm* (MPRCM 94-83) was obtained from Shiraz medicinal plants store on 25 June 2012 and identified by Dr. Moein. Voucher specimens have been deposited in the herbarium of Medicinal Plants Processing Research Center, Shiraz University of Medical Sciences, Iran. The collected plants were carefully separated, washed with distilled water, and then dried under shade for two weeks. The dried plants were crushed and grounded with a blender to obtain the powder. Condensed powders, namely, 650 g of *OP,* 415 g of *Sm*, and 522 g of *Zm*, were extracted with 99% ethanol (13 L, 8 L, and 11 L, respectively) for 1 week to obtain ethanol extracts. The extraction process was repeated three times, and the extracts were concentrated by rotary evaporator under vacuum at 40 °C.

Then, crude extracts were suspended in water 30% and methanol 70%, and then filtered. Subsequently, fractionation was done consecutively with 3 × 2000 mL petroleum ether, 3 × 1000 mL chloroform, 3 × 650 mL ethyl acetate, and 3 × 160 mL *n-*butanol.

### Enzyme inhibition procedure

Inhibition of α-glucosidase was evaluated according to the chromogenic method described by McCue et al. (2005), with some modifications. The enzyme solution contained 5 μL of α-glucosidase (25 unit/mL) and 125 μL of phosphate buffer (pH 6.9, 0.1 M). *p*-Nitrophenyl-α-d-glucopyranoside (11 mM) in the same buffer (pH 6.9) was used as the substrate solution.

Test samples (20 μL) at various concentrations were mixed with enzyme solution in microplate wells and then incubated for 15 min at 37 °C. The reaction was started by adding 20 μL of substrate solution and then incubated for an additional 15 min. The reaction was terminated by adding 80 μL of 0.2 M sodium carbonate solution.

Absorbance of the wells was measured by a microplate reader at 405 nm, while the reaction system without plant extracts was used as thec ontrol. The system without α-glucosidase was used as the blank, and acarbose was used as the positive control. All determinations were performed in triplicate. The enzyme inhibitory rates of the samples were calculated as follows:

Inhibition%= [(control absorption − sample absorption)/control absorption]* 100

### Kinetics of α-glucosidase inhibition

Inhibition modes of the samples against α-glucosidase were determined according to the method described by Kim et al. (2005). Briefly, fixed amounts of α-glucosidase were incubated with increasing concentrations of its substrates (PNPG) at 37 °C for 15 min, in the absence or presence of samples (concentration equivalent to IC_50_). Reactions were terminated, and absorption was measured and converted to reaction by Lineweaver–Burk plot. All the determinations were performed in triplicate.

### Statistical analysis

The data were expressed as the mean ± SD of three replicates. Analysis was performed using Graphpad Software and Excel 2010. One-way analysis of variance (ANOVA) and Tukey posttest were used to evaluate the possible differences among the means. *p* values ≤0.05 were considered as significant differences.

## Results

### *In vitro* α-glucosidase inhibition

**Figure 1.**
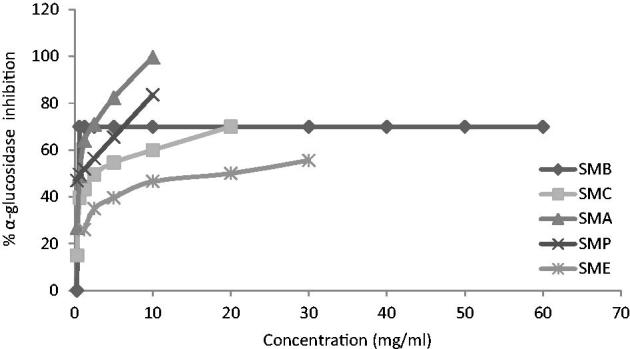
Inhibition of α-glucosidase by alcoholic extract of *Salvia mirzayanii* and its fractions at different concentrations.

**Figure 2.**
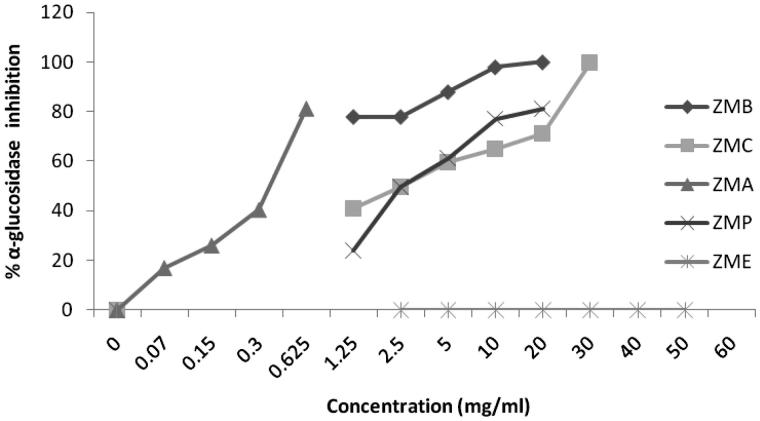
Inhibition of α-glucosidase by alcoholic extract of *Zataria multiflora* and its fractions at different concentrations.

**Figure 3.**
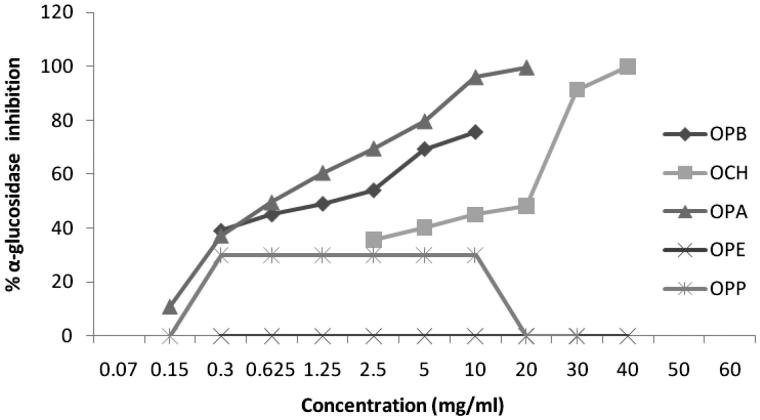
Inhibition of α-glucosidase by alcoholic extract of *Otostegia persica* and its fractions at different concentrations.

Alcoholic extracts of *Sm, Zm* and *Op* and four different fractions of them were tested for α-glucosidase inhibitory property with application of colorimetric method. Sampling information including taxonomic status, date, and location of gathering the plants is presented in Table 1. The α-glucosidase inhibition potential of three extracts as well as their fractions were compared on the basis of their IC_50_ values, which are the concentrations that produce 50% inhibition under the researchers’s specific set of assay conditions (Table 2). Ethyl acetate fraction of *Zm* (*ZMA*) exhibited the highest inhibitory effect with an IC_50_ = 0.35 ± 0.01 mg/mL in comparison with other samples and standard. Figures 1–3 indicate the enzyme inhibition percentage versus increasing concentrations of the samples.

**Table 1.** Sampling information: taxonomic status, date and location of gathering.

Phylum	Family	Genius	Date	Locality
Magnoliphyta	Lamiaceae	*Salvia mirzayanii*	20 Jun 2012	Genu Mountains
Magnoliphyta	Lamiaceae	*Otostegia persica*	15 Jun 2012	Genu Mountains
Magnoliphyta	Lamiaceae	*Zataria multiflora*	25 Jun 2012	Nearest of Shiraz

**Table 2.** α-Glucosidase inhibitory potential (IC_50_) of the different samples (mg/ml) (Positive control: acarbose (IC_50_ = 11 ± 0.19 mM∼7 mg/ml).

Samples	IC_50_	Samples	IC_50_	Samples	IC_50_
SMP	0.4 ± 0.11	ZMP	2.5 ± 0.03	OPP	ND
SMC	4.5 ± 0.43	ZMC	2.51 ± 0.23	OPC	10 ± 0.51
SMA	0.8 ± 0.22	ZMA	0.35 ± 0.01	OPA	0.5 ± 0.16
SMB	ND	ZMB	ND	OPB	1.25 ± 0.22
SME	17 ± 0.26	ZME	0	OPE	0

Values are mean of three replicate determinations (*n* = 3) ± standard deviation.

ND: None determined.

P: petroleum ether fraction; C: chloroform fraction; A: ethyl acetate fraction; B: butanol fraction; E: ethanol extract.

### α-Glucosidase kinetic studies

**Figure 4.**
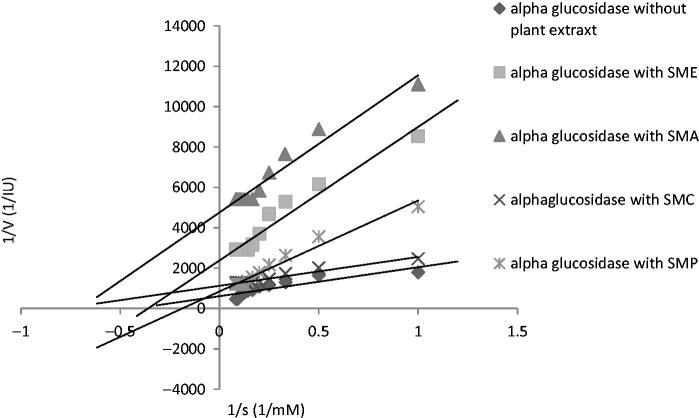
Kinetic analysis of-glucosidase inhibition by different fractions of *Salvia mirzayanii* extract.

**Figure 5.**
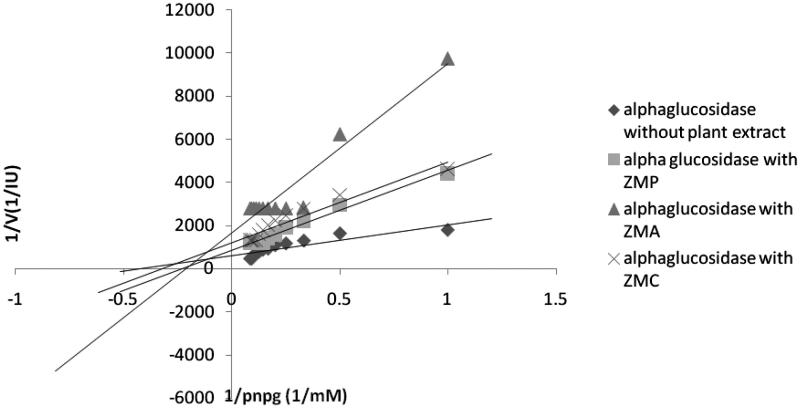
Kinetic analysis of α-glucosidase inhibition by different fractions of *Zataria multiflora* extract.

**Figure 6.**
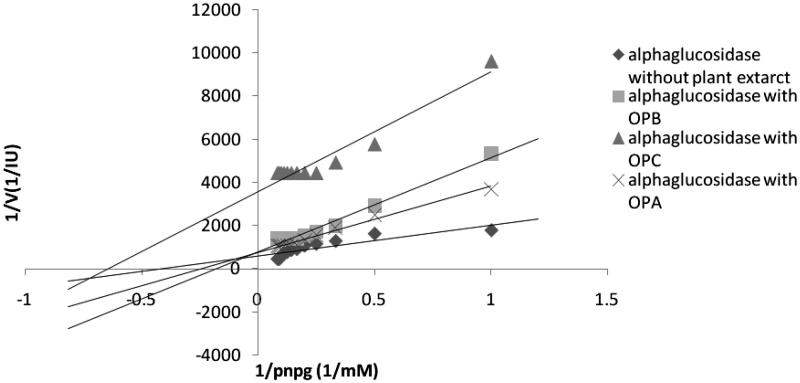
Kinetic analysis of α-glucosidase inhibition by different fractions of *Otostegia persica* extract.

To characterize the inhibition process, i.e., competitive, noncompetitive, uncompetitive, or mixed, data are often analyzed by those set of techniques that linearize inherently non-linear relationships. One of the techniques is the Linewaver–Burk kinetic analysis. Table 3 demonstrates the *K*_m_ and *V*_max_ values of the extracts against α-glucosidase. As compared with the uninhibited reaction, a decrease in *V*_max_ was noted for all tested extracts; however, the effects of the extracts on *K*_m_ were different, which means that *K*_m_ values were reduced in presence of chloroform fraction of *Zm* ethanol extract (*ZMC*), chloroform fraction of *Sm* ethanol extract (*SMC*), ethyl acetate fraction of *Sm* ethanol extract (*SMA*), *Sm* ethanol extract (*SME*), and chloroform fraction of *Op* ethanol extract (*OPC*) and increased in presence of *ZMA*, petroleum ether fraction of *Zm* ethanol extract (*ZMP)*, petroleum ether fraction of *Sm* ethanol extract (*SMP*), ethyl acetate fraction of *Op* ethanol extract of (*OPA*) and *n*-butanol fraction of *Op* ethanol extract (*OPB*) (Table 3). It is the first time that the inhibition mechanisms of α-glucosidase by *Sm*, *Op*, and *Zm* different fractions of ethanolic extracts were investigated according to these patterns (Figures 4–6). The results showed that all of the samples may display patterns of mixed inhibition as uncompetitive-noncompetitive inhibitions.

**Table 3.** Kinetic parameters of α-glucosidase inhibition by plant extracts and their different fractions.

Plant extracts	*K*_m_ mM	*V*_max_ μM^.^min^−1^
α-Glucosidase without inhibitor	2.29 ± 0.005	0.0016 ± 0.0000
*Salvia mirzayanii* ethyl acetate fraction	1.36 ± 0.001	0.0002 ± 0.00001
*Salvia mirzayanii* petroleum ether fraction	5.4 ± 0.005	0.0012 ± 0.00006
*Salvia mirzayanii* chloroform fraction	1.28 ± 0.006	0.0009 ± 0.00002
*Salvia mirzayanii* ethanol extract	2.64 ± 0.003	0.0004 ± 0.00002
*Zataria multiflora* ethyl acetate fraction	5.81 ± 0.005	0.0007 ± 0.00001
*Zataria multiflora* petroleum ether fraction	4.51 ± 0.002	0.0012 ± 0.00004
*Zataria multiflora* chloroform fraction	2.99 ± 0.005	0.0008 ± 0.00002
*Otostegia persica* chloroform fraction	1.66 ± 0.004	0.0003 ± 0.00001
*Otostegia persica* butanolic fraction	5.7 ± 0.002	0.0013 ± 0.00003
*Otostegia persica* ethyl acetate fraction	3.99 ± 0.003	0.0013 ± 0.00002

## Discussion

There is a rising interest among researchers to specify new and effective α-glucosidase inhibitors with minimum side effects, which can be obtained from medicinal plants with acknowledged and scientifically proven antidiabetic properties (Grover et al. 2002; Onal et al. 2005; Bnouham et al. 2006). The present study investigated the inhibitory effects of *Sm, Zm*, and *Op* ethanol extracts as well as their four different fractions on α-glucosidase. Approximately, all of the samples, with exception of *Zm* ethanol extract (*ZME*) and ethanol extract *Op* (*OPE*), revealed inhibitory effects on α-glucosidase. This finding about *ZME* is in agreement with results presented in another study conducted by Gholamhoseinian et al. (2008), which showed that *Zm* methanol extract had no effect on α-glucosidase inhibition. In three geniuses, inhibition effects of ethanol extracts were less than those of their fractions, which might be due to interactions taking place between compounds in the crude extracts. This finding was in contrast with the already reported results (Chen et al. 2013).

The IC_50_ of the three samples including *SMB*, *ZMB*, and *OPP* could not be determined since inhibition percentage of these samples was not dependent on the concentrations (Table 2). It means that by increasing the concentration, no increase was observed in inhibition. Moreover, at different concentrations of, petroleum ether fraction of *Op* ethanol extract (*OPP*), *n*-butanol fraction of *Sm* ethanol extract (*SMB*), and *n*-butanol fraction of *Zm* ethanol extract (*ZMB*), just 80%, 70%, and 30% inhibition were observed, respectively. The mentioned finding has been reported in another study, which examined α-amylase inhibitory effects of six *Salvia* species and reported no relationship between the concentration level and inhibition. In other words, as the concentrations of *Salvia hydrangea*, S*alvia hypoleuca*, and S*alvia officinalis* extracts increased, the enzyme inhibition presented no increase (Nickavar et al. 2010). In the present study, the samples with determination IC_50_ were categorized into 3 groups. Group 1 included *ZMA*, *SMP*, *OPA* and *SMA*, which exhibited very noticeable inhibition activities, and their inhibitory IC_50_ values were much less than acarbose as the standard (*p* < 0.001). Furthermore, there were not significant differences between their IC_50_ values (Table 2, *p* > 0.05). Group 2 included *OPB*, *SMC*, *ZMP*, and *ZMC*, which showed remarkable inhibition activities. Their IC_50_ values were less than acarbose (Table 2, *p* < 0.001); however, it must be mentioned that IC_50_ values were not less than those of the previous group. Moreover, significant differences were observed in their IC_50_ values (*p* < 0.01). Other researchers reported that inhibition of α-glucosidase by some plant extracts such as *Susongsaengi-susu*, *Sikyung-susu*, and *Quercus brantii* extracts (Azemi et al. 2015) is more than acarbose. Group 3 included *OPC*, whose IC_50_ value was not significantly different from that of the standard (*p* > 0.05) and *SME*, whose IC_50_ value was more than that of the standard (Table 2, *p* < 0.001). This finding is agreement with results presented in another study,which examined species of *Salvia* containing three flavonoids, namely, luteolin 7-*O*-glucoside, luteolin 7-*O*-glucuronide, and diosmetin 7-*O*-glucuronide. These compounds were isolated and showed inhibitory effects on α-glucosidase with IC_50_ values close to that of acarbose as the standard (Asghari et al. 2015). Phytochemical screening of the active components of *OP* species have been revealed that several types of diterpenoids and flavonols including morin, kaempferol, and quercetin are identified in this plant; moreover, two compounds such as morin and quercetin are responsible for antioxidant activities of *OP* methanol extract; therefore, these compounds may inhibit the α-glucosidase.

In addition to, components such as catechine and rosmarinic acid were separated from *Salvia* extracts by HPLC analysis (Asadi et al. 2011).

Also, in other research, in *SMA*, chemical components such as apigenin and kaempferol were identified (the data was not published) which these components may be responsible for inhibiting the activity of α-glucosidase. The essential oil of *Zm* was analyzed by GC/MS and the major compounds were thymul (26.32%) and carvacrol (25.51%); (Emamjomeh et al. 2014); moreover, three known compounds, dihydroxy aromadendrane, luteolin and α-tocopherol quinone, have also been isolated from *Zm* extract (Ali et al. 2000). In this study, three geniuses of Labiatae revealed very significant inhibitory effects on α-glucosidase (Figures 1–3), and it seems that they enjoyed strong antidiabetic activities. Hence, with due attention to the antidiabetic compounds in the natural products (Arif et al. 2014; Benalla et al. 2010), these three plant extracts may contain antidiabetic compounds such as polyphenols, triterpenes, phytosterols, flavonoids, flavanones, genine derivatives, and anthocyanin (Ali et al. 2000; Lu & Foo 2002; Yassa et al. 2005; Sadeghi & Alizadeh 2013; Sajed et al. 2013; Sadeghi et al. 2014; Zarshenas & Krenn 2015).

Results from Lineweaver-Burk plots demonstrated that inhibition modes of all samples may be mixed inhibitions. Mixed inhibition may result in either a decrease in the apparent affinity of the enzyme for the substrate (*K*_m_^app^> *K*_m_), which means that *K*_m_ value appears to increase in cases where the inhibitor favors binding to the free enzyme, or an increase in the apparent affinity (*K*_m_^app^< *K*_m_), which means that *K*_m_ value appears to decrease when the inhibitor binds favorably to the enzyme–substrate complex. It is probable that the ability to bind to wide regions of the enzyme other than the active site enables these extracts as mixed inhibitors to reveal a broader specificity of inhibition compared with acarbose as a competitive inhibitor (Zhang et al. 2014).

One advantage of these extracts over acarbose is that in contrast with acarbose as a competitive inhibitor, these extracts (mixed inhibitors) may not be affected by higher concentrations of the substrate. It has been reported that with higher carbohydrate food intake, higher concentrations of acarbose as a competitive inhibitor would be required to present the same effect; however, with respect to mixed inhibition, the inhibitor would be still effective at lower concentrations (Ghadyale et al. 2011; Zhang et al. 2014).

As the association between phenolic compounds and inhibition of carbohydrate hydrolyzing enzymes has been revealed (Shobana et al. 2009), it seems that these samples’s mode of inhibition is due to presence of high levels of flavonoids compounds (Tadera et al. 2006; Zhang et al. 2014).

Researchers have described the following characteristics of α-glucosidase inhibitors (Azemi et al. 2015): (1) sugar (substrate)-mimic structures, (2) potency to establish ionic bonds with nucleophilic catalyzing residues, (3) transition state-like structures, (4) potency to construct hydrogen bonds with catalytic acid residues, (5) potency to construct ionic and hydrophobic interactions at sites other than the active site, and (6) potency to construct covalent bond with enzymes through an epoxy or aziridine group (Moorthy et al. 2012).

Further studies should be directed towards chemical isolation, purification, and characterization of these three extracts to elucidate the compounds responsible for inhibition potential as these may play significant role in the development of antidiabetic agents.

## Conclusions

The results of this study could offer a basis for further investigation and isolation of active compounds with α-glucosidase inhibition from the *Op*, *Sm*, and *Zm* extracts. The knowledge about the mechanisms of inhibition by these extracts could lead to affluent application of plant chemicals as drug targets.
